# Supplementary material for: A network dynamical simulation model for the study of antibiotic resistance in microbial communities
Source: Gut Microbes. 2026 Sep 19;18(1):2734703. doi: 10.1080/19490976.2026.2734703 (PMC13596919; doi:10.1080/19490976.2026.2734703)
Supplement: Supplementary file 1 [file KGMI_A_2734703_SM9053.zip › supplementary_file_1/Figures/er_delta_n_components_n100.pdf]

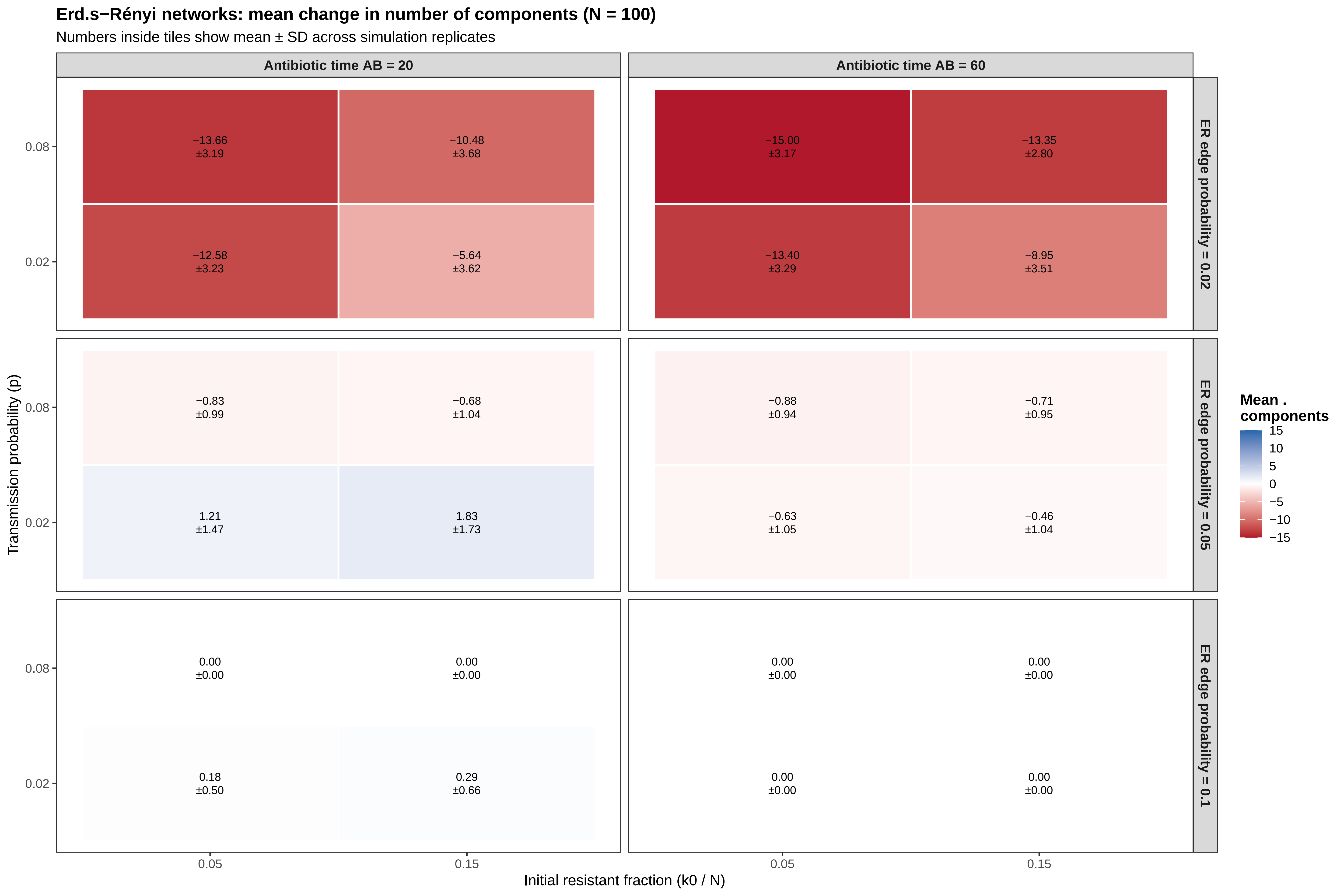
Erd.s−Rényi networks: mean change in number of components (N = 100)
Numbers inside tiles show mean ± SD across simulation replicates
Antibiotic time AB = 20
Antibiotic time AB = 60
ER edge probability = 0.02
ER edge probability = 0.05
ER edge probability = 0.1
−13.66 ±3.19
−10.48 ±3.68
−12.58 ±3.23
−5.64 ±3.62
−15.00 ±3.17
−13.35 ±2.80
−13.40 ±3.29
−8.95 ±3.51
−0.83 ±0.99
−0.68 ±1.04
1.21 ±1.47
1.83 ±1.73
−0.88 ±0.94
−0.71 ±0.95
−0.63 ±1.05
−0.46 ±1.04
0.00 ±0.00
0.00 ±0.00
0.18 ±0.50
0.29 ±0.66
0.00 ±0.00
0.00 ±0.00
0.00 ±0.00
0.00 ±0.00
Transmission probability (p)
0.08
0.02
Initial resistant fraction (k0 / N)
0.05
0.15
Mean . components
15
10
5
0
−5
−10
−15
